# Interrelationships and Shared Variance Among Three Field-Based Performance Tests in Competitive Youth Soccer Players

**DOI:** 10.3390/jfmk11010058

**Published:** 2026-01-29

**Authors:** Andrew D. Fields, Matthew A. Mohammadnabi, Oleg A. Sinelnikov, Michael R. Esco

**Affiliations:** Department of Kinesiology, The University of Alabama, Tuscaloosa, AL 35487, USA; adfields1@crimson.ua.edu (A.D.F.);

**Keywords:** youth soccer, change of direction, explosive power, aerobic capacity, counter-movement jump, field-based testing, athletic performance

## Abstract

**Objectives:** Field-based testing is commonly used to evaluate key physical qualities related to soccer performance. However, limited research has examined the degree of shared variance among measures of aerobic capacity, change of direction (COD), and explosive power in youth athletes. This study investigated the relationships between the 20 m shuttle run (20MSR), T-test (TT), and vertical countermovement jump (CMJ) to determine their unique and overlapping contributions to each other’s performance in competitive youth soccer players. **Methods:** Twenty-five competitive male youth soccer players (13.7 ± 0.8 years) completed standardized assessments of TT, CMJ, and 20MSR during pre-season evaluations. Pearson correlations and hierarchical multiple regression analyses were used to examine associations and independent variance explained among the performance measures. **Results:** Large, significant correlations were observed between TT and CMJ (r = −0.65, *p* < 0.001), TT and 20MSR (r = −0.59, *p* < 0.001), and CMJ and 20MSR (r = 0.53, *p* = 0.007). CMJ explained 42.3% of TT variance, whereas adding 20MSR did not significantly improve model fit (ΔR^2^ = 0.087, *p* = 0.062). Across models, aerobic capacity did not contribute significant unique variance beyond neuromuscular performance. **Conclusions:** COD and lower-body power share a common physiological foundation in youth soccer athletes, while aerobic capacity represents a distinct performance domain. When field tests are administered under applied conditions typical of youth soccer environments, TT and CMJ demonstrate substantial shared variance, whereas 20MSR remains largely independent. Therefore, the findings support the continued use of multi-modal testing batteries in practice.

## 1. Introduction

Identifying the key physiological attributes that contribute to soccer performance has been a central focus of sport science research for several decades [1,2,3]. Indeed, numerous investigations have demonstrated that soccer players possess a broad range of physical qualities, with certain parameters consistently emerging as critical determinants of performance in the sport [4,5,6]. Among these, change of direction (COD), lower-body explosive power (LBP), and aerobic endurance have emerged as important factors related to match performance [3]. COD plays a fundamental role in enabling athletes to have the ability to rapidly change direction, evade defenders, or effectively respond to unpredictable movements and ball trajectories during match play [7,8,9]. LBP supports quick and decisive movements that are vital to the dynamic nature of the sport, such as sprinting, jumping, and burst acceleration [8,9]. Furthermore, optimal aerobic fitness plays a foundational role in game success, supporting the ability to sustain high-intensity efforts and to recover efficiently between repeated bouts of exertion throughout the duration of a match [10,11]. Collectively, these physical attributes are key indicators of sport-specific fitness and are integral to overall soccer performance [3,4,5].

Field-based performance tests are routinely used to assess these physical fitness parameters among soccer players due to their practicality, cost-effectiveness, and ecological validity [4,5]. The T-test (TT) [12], vertical countermovement jump (CMJ) [13], and 20 m shuttle run test (20MSR) [14,15] are widely recognized as acceptable measures of COD, LBP, and aerobic fitness, respectively [4,15,16]. Although each of these tests is uniquely designed to measure a distinct performance parameter, the complex and integrated nature of soccer performance suggests a possible degree of shared influence among their assessment domains [1,17,18]. The potential shared influence could be related to underlying physiological adaptations to training [1,3] and therefore are important to determine, as this may allow coaches and sport scientists to optimize training by knowing the degree to which performance factors are related to each other. Such knowledge may, in turn, remove undue load and decrease recovery needs by implementing strategies that train multiple related factors at once instead of focusing on each distinct performance parameter [19]. However, most of the prior research examining these relationships has relied on bivariate correlations, which do not account for the extent to which shared variance among performance tests obscures their unique contributions. Consequently, practitioners lack clarity regarding how much information one test provides about another when multiple assessments are included within the same battery. Hierarchical regression offers a practical analytical approach to quantify both shared and independent variance among field-based performance tests, thereby addressing this methodological gap.

Understanding the relationships between performance indicators is particularly important among youth athletes who are rapidly developing the foundational physical qualities that underpin success in the sport. The significant changes in body composition, muscular strength, and neuromuscular coordination that occur during this stage of life can influence both sport-performance and the degree of carryover between training adaptations of specific physical qualities [1,2,7]. Therefore, identifying the overlapping qualities among these fitness tests can help coaches and practitioners streamline testing protocols and effectively tailor training interventions to developmental needs [20,21,22]. Because most testing in youth soccer occurs in applied field settings, there is a need to quantify how commonly used performance tests relate to one another when administered under real-world conditions using field-based, accessible tools. However, despite the practical implications, limited research has examined the degree of shared variance among these field-based tests, particularly among youth soccer players. Therefore, the purpose of this study was to examine the degree of shared variance among the TT, CMJ, and 20MSR performances in competitive male youth soccer players. It was hypothesized that TT and CMJ would demonstrate substantial shared variance due to common neuromuscular determinants, whereas 20MSR performance would contribute minimal unique variance to TT or CMJ after accounting for their shared relationship.

## 2. Materials and Methods

### 2.1. Participants

Data from a convenience sample of twenty-five competitive male youth soccer players (age = 13.7 ± 0.8 years, height = 167.4 ± 9.7 cm, weight = 57.6 ± 12.1 kg) were retrospectively analyzed for this study. Participants were U14 male soccer players competing in a regional youth league. Athletes trained three times per week within an organized, tryout-based competitive club environment and were selected through a state Olympic Development Program (ODP). Based on their competitive context and training exposure, players were classified as competitive youth soccer players in the specializing stage of development. Participants were eligible for inclusion if they were active members of the team and able to complete all performance assessments. Players were excluded from testing if they reported a current musculoskeletal injury, illness, or medical condition that would limit maximal effort or full participation at the time of data collection. Because this study involved a retrospective secondary analysis, additional medical screening data beyond standard team participation clearance were not available.

Although the same cohort of youth soccer players was used in a previous publication, the prior analysis focused solely on the independent relationships between body composition and the three performance tests [23]. In contrast, the present investigation focuses on quantifying the relative shared and unique variance among the three performance tests within a single cohort of competitive youth soccer players, independent of body composition influences. Although relationships among these tests have been reported previously, few studies have examined their hierarchical contributions to one another using regression-based approaches in this population, thereby providing practically relevant information for optimizing field-based testing batteries.

All testing occurred during the pre-season period, prior to the start of the competitive season. Training load performed in the days immediately preceding testing was not formally monitored. However, the participants were instructed to arrive in a normally fed and hydrated state and refrain from strenuous exercise for at least 24 h before testing. In addition, participants and their parents or legal guardians were informed of the study procedures and associated risks and benefits, and written informed consent was obtained. The original data collection was approved by the University of Alabama Institutional Review Board as part of a broader protocol supporting multiple investigations. The present secondary analysis of de-identified data did not require additional ethics review according to institutional guidelines.

### 2.2. Performance Tests

The T-test COD drill was administered on an indoor court and manually timed using handheld stopwatches operated by two trained technicians [12]. Prior to testing, participants completed an approximately 5 min dynamic warm-up consisting of repeated 20 m shuttle runs with 30 s of rest between runs. Each trial began with the participant standing with both feet behind the starting line before sprinting 9.14 m forward to touch the center cone with the dominant hand, shuffling 4.52 m to the left to touch the left cone, shuffling 9.14 m to the right to touch the right cone, and finally shuffling 4.52 m back to the center cone before backpedaling through the starting line. To minimize the potential influence of anthropometric differences on test performance, all participants were instructed to make contact with each cone using their dominant hand while maintaining an upright torso and ensuring that at least one foot crossed the vertical plane of the cone base prior to changing direction. Trials were invalidated and repeated if these criteria were not met, as judged by the test administrators. Two submaximal familiarization trials were completed, followed by three maximal-effort trials. During each trial, both technicians were positioned on either side of the starting/finishing line with an unobstructed view of the athlete. The technicians independently started and stopped their stopwatches based on the athlete’s initial movement and the torso crossing the finish line. Trial time was calculated as the mean of the two recorded stopwatch values and recorded to the nearest 0.01 s, with the fastest mean time from the three maximal trials retained for analysis. The technicians were experienced in administering the T-test and included one Certified Strength and Conditioning Specialist and one certified youth soccer coach. Although manual timing may introduce measurement error, previous research supports the validity of trained raters using handheld stopwatches for assessing sprint performance relative to electronic timing systems [24].

The CMJ was assessed indoors using a Vertec device (Questek, Sports Imports, Hilliard, OH, USA). After a brief warm-up of several submaximal squat jumps, each participant’s maximal vertical reach with their dominant hand was established. The Vertec was adjusted so the reach height was aligned with a red marker positioned 30.48 cm below the lowest vane. For each trial, players performed a countermovement jump, beginning from an upright stance with feet shoulder-width apart and using a coordinated arm swing to maximize takeoff height. Jump height was calculated as the difference between the standing reach and the highest vane displaced. Three trials were completed, and the best performance was used for analysis [13].

Aerobic capacity was evaluated using the 20MSR, completed on an outdoor soccer field. Participants ran back and forth between two cones set 20 m apart while following progressively faster audio beeps delivered through a mobile application. The speed began at 8.0 km·h^−1^, increased to 9.0 km·h^−1^ for the second stage, and then rose in 0.5 km·h^−1^ increments each minute. The test concluded when a participant failed to reach the designated line on two consecutive cues [14,25,26]. The total distance covered (meters) was recorded as the performance score. Small groups (4–8 players) completed the test together to promote motivation, and all athletes were familiar with the protocol due to regular use in training. Environmental conditions (e.g., temperature and wind) were not recorded during outdoor testing and should be considered when interpreting variability in 20MSR performance.

The CMJ and T-test were administered on the same testing day in no predetermined order, with approximately 10 min of passive recovery provided between tests. The testing order was counterbalanced across participants to minimize potential order effects. The 20MSR was performed on a separate day on an outdoor soccer field one week prior to the CMJ and T-test to avoid fatigue-related interference with the neuromuscular assessments.

### 2.3. Statistics

A priori power analysis was conducted using G*Power 3.1 software [27]. Assuming a moderate effect size for bivariate correlations (r = 0.50), α = 0.05, and power (1 − β) = 0.80, the required sample size was estimated to be 24 participants. Therefore, the sample of twenty-five athletes in the present study was deemed sufficient to detect moderate-to-large associations among the performance variables. Statistical analyses were performed using SPSS version 29.0 (IBM Corp., Chicago, IL, USA). All data were first screened for normality and potential outliers. Variables were assessed for skewness and kurtosis, with values greater than 2 indicating a non-normal distribution. No outliers were identified or removed from the dataset.

Mean ± standard deviation (SD) and minimal-to-maximal ranges were calculated for each of the three performance tests. Bivariate correlations were conducted using Pearson’s correlation coefficient (r) to evaluate the strength and direction of the relationships among the performance measures. Correlation coefficients were qualitatively interpreted using Hopkins’ scale as follows: r = 0.00–0.10 as trivial; 0.10–0.30 as small; 0.30–0.50 as moderate; 0.50–0.70 as large; 0.70–0.90 as very large; and >0.90 as near perfect [28].

To examine the shared and unique variance between the performance tests, hierarchical multiple regression analyses were conducted with each performance test entered as the dependent variable and the remaining two tests included as independent variables. In each regression, the independent variable demonstrating the strongest bivariate association with the dependent variable was entered in the first block, followed by the second independent variable in the subsequent block to evaluate any significant increase in explained variance (ΔR^2^). Standardized beta coefficients (β) and *p*-values were reported to determine the unique contribution of each variable in the final model. The alpha level of significance was predetermined as *p* < 0.05 for Pearson’s r values and the outcome variables of the regression models (i.e., R^2^ and ΔR^2^). Assumptions for multiple regression were evaluated for multicollinearity using tolerance and variance inflation factors (VIF). Independence of errors was evaluated using the Durbin–Watson statistic, and influential observations were screened using Cook’s distance.

## 3. Results

All twenty-five participants successfully completed the testing protocol. The mean scores (represented as mean ± SD) and ranges for the TT, CMJ, and 20MSR are presented in Table 1.

**Figure 1:**
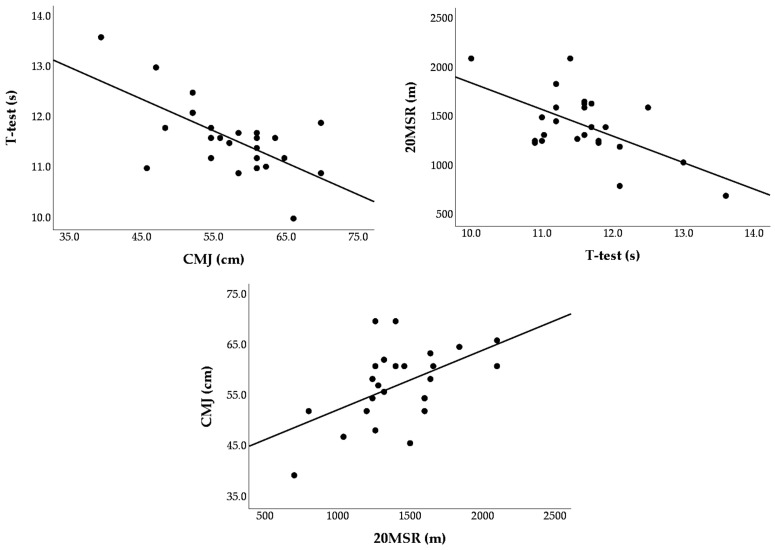
Scatterplots displaying the relationships between the performance test results. Each dot represents an individual participant. Solid lines indicate the line of best fit illustrating the direction of the association between variables.

Pearson correlation procedures revealed significant coefficients between the TT and CMJ (r = −0.65, *p* < 0.001, “large”), between the TT and 20MSR (r = −0.59, *p* = 0.002, “large”), and between the CMJ and 20MSR (r = 0.53, *p* = 0.007, “large”). Scatterplots illustrating these relationships are presented in Figure 1. Visual inspection of the scatterplots indicated clear linear trends between each pair of performance tests, with no evident non-linear patterns or heteroscedasticity. The relationships were characterized by moderate dispersion around the line of best fit and no discernible clustering or outlying cases, supporting the use of Pearson correlation and hierarchical multiple regression procedures.

The results of the hierarchical multiple regression procedures are shown in Table 2. When TT was the dependent variable, CMJ alone explained 42.3% of the variance (*p* < 0.001). Adding 20MSR increased the explained variance to 50.9%, but this change was not significant (*p* = 0.062). Similarly, when CMJ was the dependent variable, TT alone accounted for 42.3% of the variance (*p* < 0.001). However, the inclusion of 20MSR, which increased explained variance to 45.3%, did not significantly improve the model (*p* = 0.282). For 20MSR, TT initially explained 35.0% of the variance (*p* = 0.002). The addition of CMJ did not significantly increase the explained variance above and beyond that of TT alone (*p* = 0.282).

Regression diagnostics indicated acceptable model assumptions across all hierarchical regression models. No influential observations were identified (maximum Cook’s distance = 0.43). Multicollinearity was not evident among predictor variables in any model (tolerance range = 0.58–0.73; VIF range = 1.38–1.73). Independence of residuals was acceptable for TT (Durbin-Watson = 2.15), CMJ (Durbin-Watson = 1.78), and 20MSR (Durbin-Watson = 2.01).

## 4. Discussion

The purpose of this study was to examine the interrelationships and degree of shared variance among the T-test, vertical countermovement jump, and 20 m shuttle run field tests. These tests were selected due to their individual assessments of important factors related to soccer performance (i.e., COD, LBP, and aerobic capacity), as well as their widespread use, particularly among youth soccer players [6,9,26]. The results revealed strong bivariate correlations among all three performance tests (range = 0.53 to −0.65). However, the hierarchical regression analyses demonstrated that only TT and CMJ meaningfully explained each other’s variance (R^2^ = 0.423, *p* < 0.001), whereas TT was the sole significant independent variable accounting for the variance of 20MSR performance (R^2^ = 0.350, *p* = 0.002). Although 20MSR was correlated with both TT and CMJ, it did not contribute significant unique variance to either outcome, nor did CMJ significantly account for the variance in 20MSR. These findings provide insight into the hierarchical nature of movement capabilities and the extent to which COD, lower-body power, and aerobic performance overlap in competitive youth soccer players.

The large and independent associations between TT and CMJ suggest that participants who demonstrated more efficient COD movements also tended to possess greater vertical power capabilities, and vice versa. Similar associations between CMJ performance and agility-related outcomes have been reported across athletic populations, suggesting partial overlap among power and COD ability [9,11,12]. In addition, significant relationships between jump performance and agility measures have been documented in youth football players using comparable field-based assessments, supporting the relevance of the present findings within a developmental context [9,29,30,31]. Consistent with the hierarchical regression results observed here, once the shared variance between TT and CMJ was accounted for, the inclusion of 20MSR did not explain additional unique variance, indicating that aerobic performance contributes little independent information to these neuromuscular measures. Collectively, these characteristics help explain why athletes with greater explosive force production tend to navigate directional changes with greater efficiency and reduced transition time.

Despite these shared characteristics, the association between TT and CMJ was not perfect, possibly explained by physical and psychological attributes that are not shared between the two tests. COD movements occur predominantly in the horizontal plane and require substantial cognitive–perceptual processing, including spatial awareness and movement anticipation [12,32,33]. In contrast, vertical jumping is executed in a primarily vertical plane and is performed in a predictable, self-paced context that likely lowers cognitive demand [13]. COD testing also places greater demands on multi-directional braking, trunk control, and reactive limb stiffness, whereas CMJ emphasizes maximal force production with minimal stabilization requirements [3,8,9,10]. These distinctions are likely to contribute to the unexplained variance between the two assessments. As such, the findings reinforce that TT and CMJ do not represent separate qualities but rather reflect overlapping yet distinct sport-specific tasks. Practitioners may therefore consider using both tests to obtain a more complete profile of lower-body explosiveness across different planes of motion.

Although all three performance tests were significantly correlated, the 20MSR did not contribute uniquely to TT or CMJ after accounting for their shared variance. The 20MSR is highly dependent on maximal oxygen uptake and aerobic metabolism during its progressive stages, qualities that are not strongly linked to the short-duration, high-force actions required in TT and CMJ [26]. This distinction supports the interpretation that aerobic capacity represents a separate physiological construct from the more similar anaerobic nature of COD or explosive power [3,10]. While athletes with superior aerobic fitness may experience better fatigue resistance during repeated high-intensity efforts, single-trial tests such as TT and CMJ do not impose sufficient duration or metabolic demand to meaningfully engage aerobic pathways [4,5,11]. As such, the observed correlations between 20MSR and the two other field tests may reflect general athleticism rather than direct physiological interdependence. This interpretation is consistent with research indicating that endurance training alone does not substantially improve COD or explosive power unless combined with strength or plyometric training [1,34].

When 20MSR was modeled as the dependent variable, TT remained the only significant independent variable. This may be partially explained by methodological and biomechanical similarities between the two tests. Both involve repeated short-distance shuttles with rapid turns in opposite directions. Although the 20MSR is considered a marker of aerobic capacity, its intermittent stops, 180° turns, and reacceleration require substantial anaerobic contribution and efficient change-of-direction mechanics [11]. Athletes who demonstrate economical directional transitions during TT may therefore also maintain greater running efficiency during the 20MSR. In contrast, the CMJ measures maximal vertical power in a single, linear action with no directional change or repeated locomotion pattern [13]. As a result, its correlation with the 20MSR was largely explained by the contribution of TT, rather than a unique relationship between CMJ and aerobic capacity.

From a practical standpoint, these findings provide quantitative support for the continued use of multi-modal field-based testing batteries in competitive youth soccer players. Rather than introducing new conceptual principles, the present data offer applied confirmation of the extent to which TT and CMJ share common variance, while 20MSR remains largely independent when assessed using commonly implemented field tests. For example, when time or equipment availability is limited during pre-season testing, practitioners seeking to prioritize neuromuscular profiling may retain both the TT and CMJ to capture complementary information related to explosive power and change-of-direction performance, whereas aerobic fitness should be assessed using the 20MSR rather than inferred from neuromuscular tests. By quantifying these relationships within a single homogeneous cohort using procedures routinely employed in applied settings, this study provides practitioners with empirical justification for retaining both neuromuscular and aerobic assessments when profiling developing athletes.

Several limitations should be acknowledged when interpreting the findings of this study. Although the study was adequately powered to detect moderate-to-large effects, the relatively small sample size and inclusion of only male competitive youth soccer players may limit generalizability to other age groups, competition levels, or female athletes. Training load performed in the days preceding testing was not objectively monitored, which limits the ability to fully account for the potential influence of residual fatigue on performance outcomes. Additionally, although two trained technicians and averaged stopwatch values were used, the manual timing of the T-test may have introduced measurement error. The TT assessed pre-planned directional changes and therefore did not capture the perceptual–cognitive demands associated with reactive agility commonly observed in match play. Furthermore, biological maturation status (e.g., peak height velocity offset) was not assessed, which is relevant at this age and may influence the relationships among COD, power, and aerobic performance. In addition, environmental conditions during the outdoor 20MSR (e.g., wind and temperature) were not documented, which may have introduced additional measurement variability. Finally, the cross-sectional design precludes causal inference and does not allow determination of whether training-induced changes in one performance domain translate to improvements in the others over time.

## 5. Conclusions

In conclusion, although all three tests were all associated with one another, lower-body anaerobic fitness emerged as the primary shared contributor to performance outcomes involving LBP and COD. Aerobic fitness appears to contribute a separate and distinct component of physical fitness that is not accurately accounted for using directional speed or power assessments alone. Collectively, these findings support the integration of multiple performance tests when evaluating comprehensive athletic ability in competitive youth soccer players and emphasize the importance of selecting tools that align directly with the physiological attributes the practitioner intends to measure or train. However, given the relatively small sample size and inclusion of only male athletes from a single competitive program, these results should be generalized cautiously and primarily to competitive male youth soccer players of similar age and training background.

## Figures and Tables

**Table 1:** Mean ± standard deviation (SD) and minimum (Min) and maximum (Max) values for the performance variables.

Variable	Mean ± SD	Min	Max
TT (s)	11.6 ± 0.7	10.0	13.6
CMJ (cm)	57.3 ± 7.4	39.4	70.0
20MSR (m)	1418.4 ± 331.6	700	2100

TT = T-test, CMJ = vertical countermovement jump, 20MSR = 20 m shuttle run test.

**Table 2:** Hierarchical multiple regression results showing Block 1 and Block 2 entry for each dependent variable.

Dependent Variable	Block	IV (s)	R^2^	∆R^2^	β	*p*
TT	1	CMJ	0.423	-	−0.650	<0.001
	2	CMJ + 20MSR	0.509	0.087	−0.345	0.062
CMJ	1	TT	0.423	-	−0.650	<0.001
	2	TT + 20MSR	0.453	0.030	0.216	0.282
20MSR	1	TT	0.350	-	−0.434	0.002
	2	TT + CMJ	0.384	0.034	0.243	0.282

TT = T-test, CMJ = vertical countermovement jump, 20MSR = 20 m shuttle run test, IV = independent variable, R^2^ = explained variance, ∆R^2^ = change in explained variance, β = standardized beta coefficient, *p* = level of statistical significance.

## Data Availability

The original contributions presented in this study are included in the article. Further inquiries can be directed to the corresponding author.

## Abbreviations

The following abbreviations are used in this manuscript:

LBP	lower-body explosive power
COD	change-of-direction
TT	T-test
CMJ	vertical countermovement jump
20MSR	maximal 20 m shuttle run
cm	centimeter(s)
kg	kilogram(s)
m	meter(s)
s	second(s)
km∙h^−1^	kilometer(s) per hour
SPSS	Statistical Package for Social Sciences
SD	standard deviation
r	Pearson’s correlation coefficient
R^2^	variance
ΔR^2^	change in variance
Min	minimum
Max	maximum
